# Supplementary material for: Expanding Horizons in Cholangiocarcinoma: Emerging Targets Beyond FGFR2 and IDH1
Source: Int J Mol Sci. 2025 Nov 5;26(21):10755. doi: 10.3390/ijms262110755 (PMC12608419; doi:10.3390/ijms262110755)
Supplement: Supplementary file 1 [file ijms-26-10755-s001.zip › ijms-3908686-supplementary.pptx]

## Slide 1
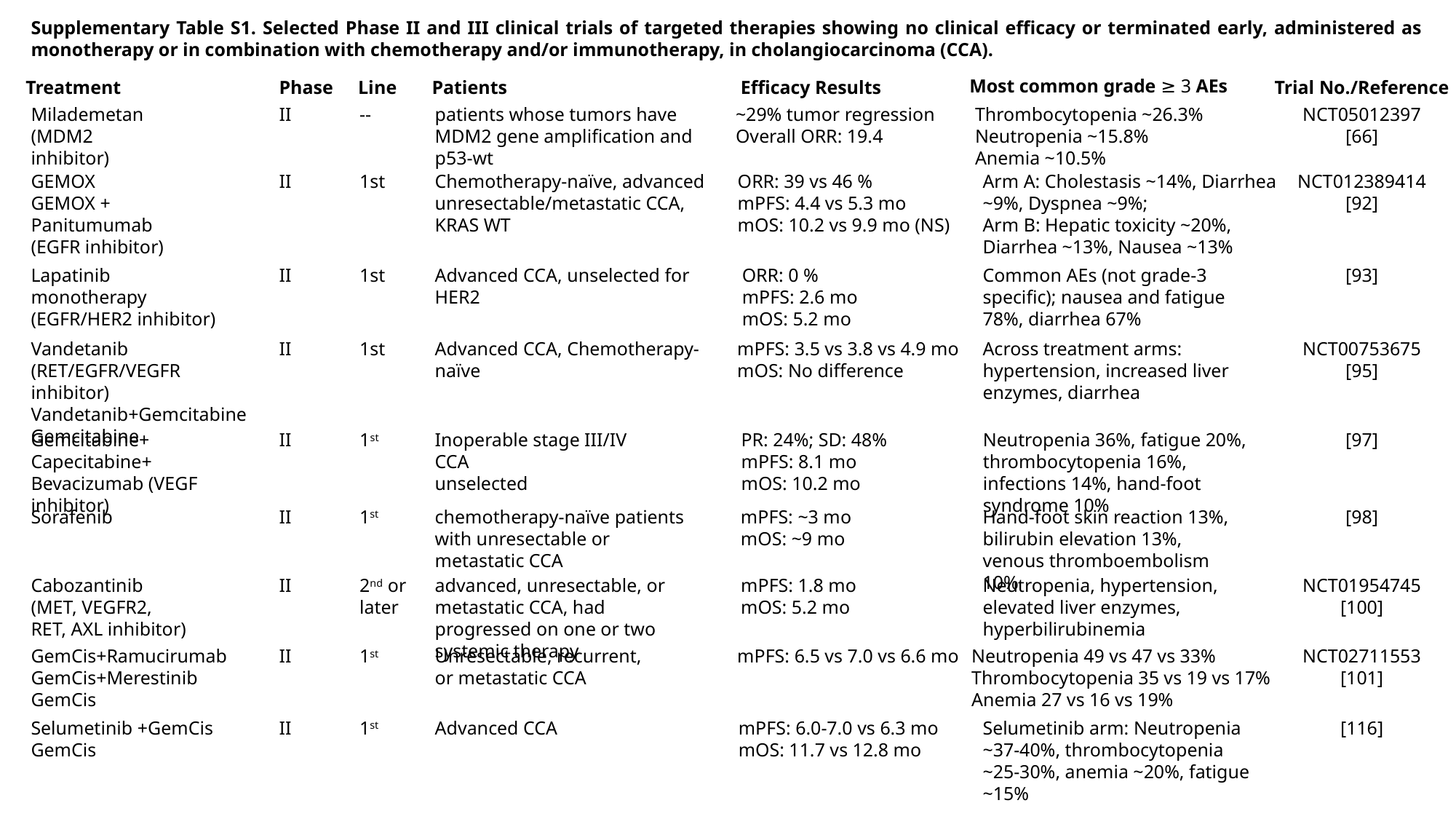

Supplementary Table S1. Selected Phase II and III clinical trials of targeted therapies showing no clinical efficacy or terminated early, administered as monotherapy or in combination with chemotherapy and/or immunotherapy, in cholangiocarcinoma (CCA).
Most common grade ≥ 3 AEs
Treatment
Phase
Line
Patients
Efficacy Results
Trial No./Reference
Milademetan (MDM2 inhibitor)
II
--
patients whose tumors have MDM2 gene amplification and p53-wt
~29% tumor regression
Overall ORR: 19.4
Thrombocytopenia ~26.3%
Neutropenia ~15.8%
Anemia ~10.5%
NCT05012397
[66]
GEMOX
GEMOX + Panitumumab
(EGFR inhibitor)
II
1st
Chemotherapy-naïve, advanced unresectable/metastatic CCA, KRAS WT
ORR: 39 vs 46 %
mPFS: 4.4 vs 5.3 mo
mOS: 10.2 vs 9.9 mo (NS)
Arm A: Cholestasis ~14%, Diarrhea ~9%, Dyspnea ~9%;
Arm B: Hepatic toxicity ~20%, Diarrhea ~13%, Nausea ~13%
NCT012389414
[92]
Lapatinib monotherapy
(EGFR/HER2 inhibitor)
II
1st
Advanced CCA, unselected for HER2
ORR: 0 %
mPFS: 2.6 mo
mOS: 5.2 mo
Common AEs (not grade-3 specific); nausea and fatigue 78%, diarrhea 67%
[93]
Vandetanib
(RET/EGFR/VEGFR inhibitor)
Vandetanib+Gemcitabine
Gemcitabine
II
1st
Advanced CCA, Chemotherapy-naïve
mPFS: 3.5 vs 3.8 vs 4.9 mo
mOS: No difference
Across treatment arms: hypertension, increased liver enzymes, diarrhea
NCT00753675
[95]
Gemcitabine+
Capecitabine+
Bevacizumab (VEGF inhibitor)
II
1st
Inoperable stage III/IV CCA
unselected
PR: 24%; SD: 48%
mPFS: 8.1 mo
mOS: 10.2 mo
Neutropenia 36%, fatigue 20%, thrombocytopenia 16%, infections 14%, hand-foot syndrome 10%
[97]
Sorafenib
II
1st
chemotherapy-naïve patients with unresectable or metastatic CCA
mPFS: ~3 mo
mOS: ~9 mo
Hand-foot skin reaction 13%, bilirubin elevation 13%, venous thromboembolism 10%
[98]
Cabozantinib
(MET, VEGFR2, RET, AXL inhibitor)
II
2nd or later
advanced, unresectable, or metastatic CCA, had progressed on one or two systemic therapy
mPFS: 1.8 mo
mOS: 5.2 mo
Neutropenia, hypertension, elevated liver enzymes, hyperbilirubinemia
NCT01954745
[100]
GemCis+Ramucirumab
GemCis+Merestinib
GemCis
II
1st
Unresectable, recurrent, or metastatic CCA
mPFS: 6.5 vs 7.0 vs 6.6 mo
Neutropenia 49 vs 47 vs 33%
Thrombocytopenia 35 vs 19 vs 17%
Anemia 27 vs 16 vs 19%
NCT02711553
[101]
Selumetinib +GemCis GemCis
II
1st
Advanced CCA
mPFS: 6.0-7.0 vs 6.3 mo
mOS: 11.7 vs 12.8 mo
Selumetinib arm: Neutropenia ~37-40%, thrombocytopenia ~25-30%, anemia ~20%, fatigue ~15%
[116]

## Slide 2
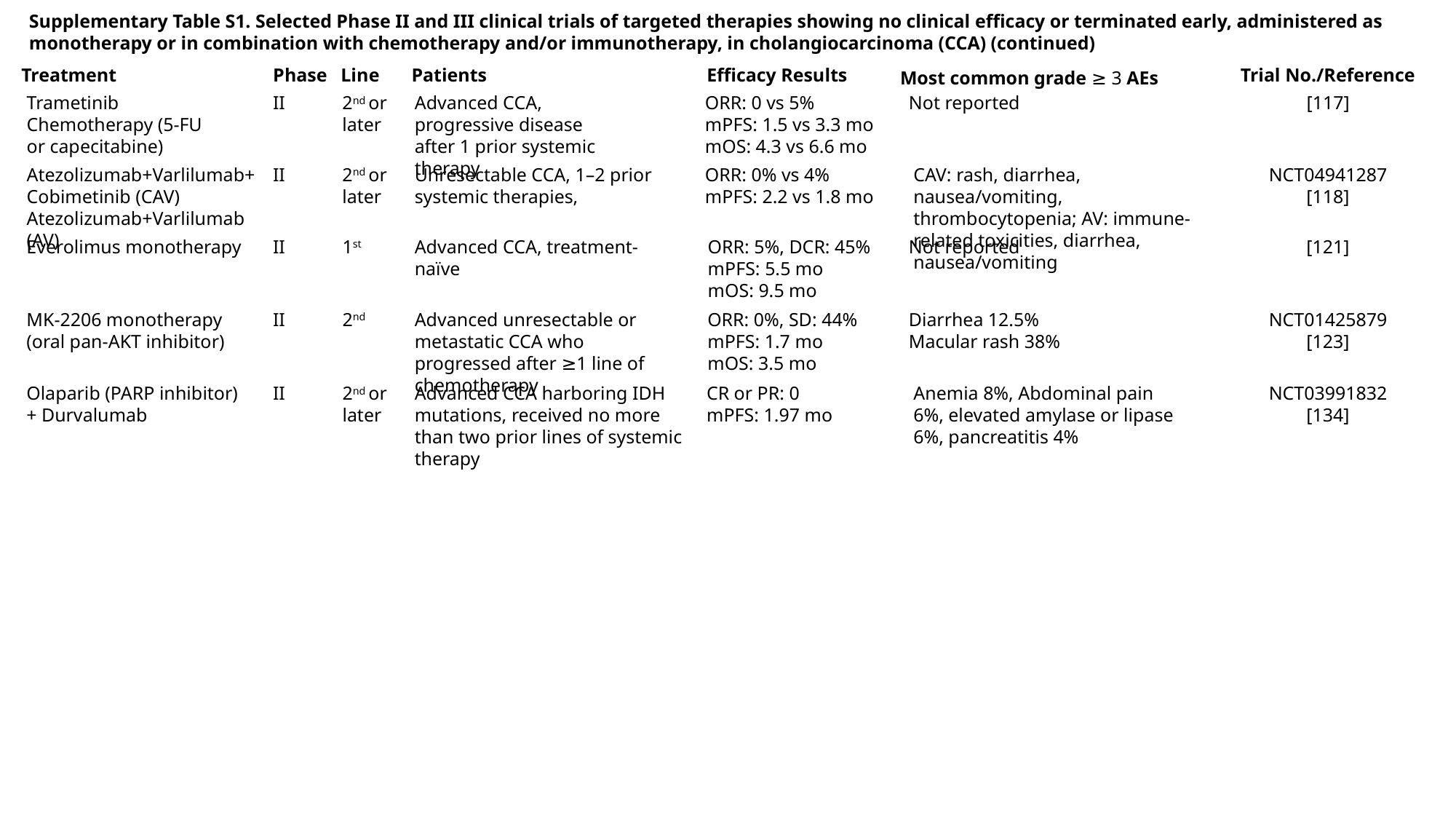

Supplementary Table S1. Selected Phase II and III clinical trials of targeted therapies showing no clinical efficacy or terminated early, administered as monotherapy or in combination with chemotherapy and/or immunotherapy, in cholangiocarcinoma (CCA) (continued)
Treatment
Phase
Line
Patients
Efficacy Results
Trial No./Reference
Most common grade ≥ 3 AEs
Trametinib
Chemotherapy (5-FU or capecitabine)
II
2nd or later
Advanced CCA, progressive disease after 1 prior systemic therapy
ORR: 0 vs 5%
mPFS: 1.5 vs 3.3 mo
mOS: 4.3 vs 6.6 mo
Not reported
[117]
Atezolizumab+Varlilumab+
Cobimetinib (CAV)
Atezolizumab+Varlilumab (AV)
II
2nd or later
Unresectable CCA, 1–2 prior systemic therapies,
ORR: 0% vs 4%
mPFS: 2.2 vs 1.8 mo
CAV: rash, diarrhea, nausea/vomiting, thrombocytopenia; AV: immune-related toxicities, diarrhea, nausea/vomiting
NCT04941287
[118]
Everolimus monotherapy
II
1st
Advanced CCA, treatment-naïve
ORR: 5%, DCR: 45%
mPFS: 5.5 mo
mOS: 9.5 mo
Not reported
[121]
MK-2206 monotherapy
(oral pan-AKT inhibitor)
II
2nd
Advanced unresectable or metastatic CCA who progressed after ≥1 line of chemotherapy
ORR: 0%, SD: 44%
mPFS: 1.7 mo
mOS: 3.5 mo
Diarrhea 12.5%
Macular rash 38%
NCT01425879
[123]
Olaparib (PARP inhibitor)
+ Durvalumab
II
2nd or later
Advanced CCA harboring IDH mutations, received no more than two prior lines of systemic therapy
CR or PR: 0
mPFS: 1.97 mo
Anemia 8%, Abdominal pain 6%, elevated amylase or lipase 6%, pancreatitis 4%
NCT03991832
[134]
